# SMOC1 colocalizes with Alzheimer’s disease neuropathology and delays Aβ aggregation

**DOI:** 10.1002/alz.090551

**Published:** 2025-01-03

**Authors:** Kaleah Balcomb, Caitlin Johnston, Tomas Kavanagh, Glenda M Halliday, Thomas Wisniewski, Margaret Sunde, Eleanor Drummond

**Affiliations:** ^1^ The University of Sydney, Sydney, NSW Australia; ^2^ New York University Grossman School of Medicine, New York, NY USA

## Abstract

**Background:**

SMOC1 has recently emerged as one of the most significant and consistent new biomarkers of early Alzheimer’s disease (AD). SMOC1 is one of the earliest changing proteins in AD, with SMOC1 cerebrospinal fluid levels increasing 29 years before symptom onset in autosomal dominant AD. Despite this clear association with disease, very little is known about the role of SMOC1 in AD or its function in the brain. Therefore, the aim of this study was to study the distribution of SMOC1 in human AD brain tissue and to determine if SMOC1 influenced Aβ aggregation.

**Methods:**

The distribution of SMOC1 in human brain tissue was assessed in 3 brain regions (temporal cortex, hippocampus, frontal cortex) using immunohistochemistry in a cohort of 74 cases encompassing advanced AD, mild cognitive impairment (MCI), preclinical AD and cognitively normal controls. The Aβ‐SMOC1 interaction was assessed in advanced AD human brain tissue (n = 6) using co‐immunoprecipitation and the influence of SMOC1 on Aβ aggregation kinetics was assessed using Thioflavin T assays and electron microscopy.

**Results:**

SMOC1 strongly colocalized with a subpopulation of amyloid plaques in AD (44.0±2.6%), MCI (33.0±5.9%) and preclinical AD (28.5±6.4%). SMOC1 levels in the brain strongly correlated with plaque load, irrespective of disease stage. Confocal imaging confirmed that SMOC1 consistently colocalized with Aβ fibrils within plaques and CAA, suggesting a possible direct interaction. SMOC1 also colocalized with a subpopulation of pTau aggregates in AD (13.2±3.1%), albeit at a much lower intensity than observed for plaques or CAA. A SMOC1‐Aβ interaction was confirmed in human AD brain tissue using co‐immunoprecipitation. Thioflavin T aggregation assays showed that SMOC1 significantly delayed Aβ aggregation in a dose‐dependent manner. Electron microscopy confirmed that the Aβ aggregates generated in the presence of SMOC1 had a different morphology; fibrils were thinner and more twisted and many small, spherical, oligomeric structures were present.

**Conclusions:**

SMOC1 colocalizes with amyloid plaques, CAA and tau pathology. SMOC1 interacts with Aβ and delays Aβ aggregation *in vitro*, making it a key candidate for future drug development studies.

